# Stochastic learning in oxide binary synaptic device for neuromorphic computing

**DOI:** 10.3389/fnins.2013.00186

**Published:** 2013-10-31

**Authors:** Shimeng Yu, Bin Gao, Zheng Fang, Hongyu Yu, Jinfeng Kang, H.-S. Philip Wong

**Affiliations:** ^1^Department of Electrical Engineering, Center for Integrated Systems, Stanford UniversityStanford, CA, USA; ^2^School of Computing, Informatics, and Decision Systems Engineering, Arizona State UniversityTempe, AZ, USA; ^3^Institute of Microelectronics, Peking UniversityBeijing, China; ^4^Institute of Microelectronics, Agency for Science, Technology and ResearchSingapore, Singapore; ^5^Department of Electrical and Electronic Engineering, South University of Science and Technology of ChinaShenzhen, China

**Keywords:** resistive switching, oxide RRAM, synaptic device, binary synapse, neuromorphic computing, stochastic learning, switching variability

## Abstract

Hardware implementation of neuromorphic computing is attractive as a computing paradigm beyond the conventional digital computing. In this work, we show that the SET (off-to-on) transition of metal oxide resistive switching memory becomes probabilistic under a weak programming condition. The switching variability of the binary synaptic device implements a stochastic learning rule. Such stochastic SET transition was statistically measured and modeled for a simulation of a winner-take-all network for competitive learning. The simulation illustrates that with such stochastic learning, the orientation classification function of input patterns can be effectively realized. The system performance metrics were compared between the conventional approach using the analog synapse and the approach in this work that employs the binary synapse utilizing the stochastic learning. The feasibility of using binary synapse in the neurormorphic computing may relax the constraints to engineer continuous multilevel intermediate states and widens the material choice for the synaptic device design.

## Introduction

In the memory hierarchy of today's von Neumann digital system, the increasing gap between the caches and the non-volatile storage devices in terms of write/read speed has become the performance bottleneck of the whole system. Bio-inspired neuromorphic computing breaks this von Neumann bottleneck because it takes the advantage of massive parallelism that comes from the distributed computing and localized storage in networks (Mead, 1990; Poon and Zhou, 2011). Neuromorphic computing is also inherently error-tolerant, thus it is especially attractive for applications such as image or speech recognition which involve a huge amount of correlated input data in a changing and indeterministic environment (Le et al., 2012). The most advanced neuromorphic computing systems today are implemented by artificial neural network in software. For example, the IBM team performed a cortical simulation at the complexity of the cat brain on Blue Gene supercomputer, which required huge amount of computation resources:147,456 microprocessors and 144 TB of memories consuming a power of 1.4 MW (Preissl et al., 2012). The parallelism of a multi-core computer pales in comparison to the highly distributed computing in 10^11^ neurons and 10^15^ synapses in the human brain (Kandel et al., 2000). As an alternative approach, the hardware implementation of neuromorphic computing may physically reproduce the parallelism on chip. Previously, neuromorphic system in hardware with both neurons and synapses was implemented by CMOS circuits (Indiveri et al., 2006). The scaling-up of these systems is mainly constrained by the device density and energy consumption of the synapses since there are thousands of synapses connecting to one neuron. And each synapse is implemented with quite a few transistors, e.g., the 8-T SRAM cells (Merolla et al., 2011) that occupies a huge area (>100*F*^2^, *F* is the minimum feature size of the lithography technology) and consumes substantial static power. Recently, two-terminal emerging memory devices that show electrically-triggered resistive switching phenomenon have been proposed as artificial synapse (Kuzum et al., 2013). These emerging memories have the advantage of a small cell area (4*F*^2^, and 4*F*^2^/m if 3D stackable, m is the number of 3D stack layer). In the literature, Ge_2_Sb_2_Te_5_ based phase change memory (Bichler et al., 2012; Kuzum et al., 2012; Suri et al., 2012a), Ag/a-Si (Jo et al., 2010), Ag/Ag_2_S (Ohno et al., 2011) based conductive bridge memory, and TiO_*x*_ (Xia et al., 2009; Seo et al., 2011), WO_*x*_ (Chang et al., 2011; Yang et al., 2012a), HfO_*x*_ (Yu et al., 2011a) based oxide resistive switching memory have been reported showing synaptic behaviors. Among these candidates, oxide based resistive switching memory is attractive for the large-scale demonstration of a neuromorphic system due to a relatively lower energy consumption (as compared to the phase change memory), the compatibility with CMOS technology and the potential for 3D integration (Wong et al., 2012; Yu et al., 2013). Mb-scale to Gb-scale prototype oxide based resistive switching memory chips have been demonstrated recently (Sheu et al., 2011; Kawahara et al., 2012; Liu et al., 2013). Therefore, a hybrid neuromorphic system with CMOS neurons and oxide resistive switching synapses integrated on top of CMOS neurons at the metal interconnect layers can be envisioned.

**Figure 1:**
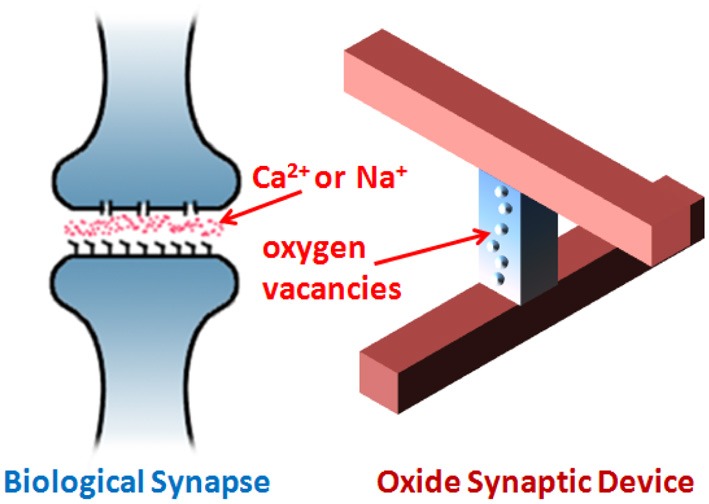
**An analogy between the biological synapse and the arifitial oxide synaptic device.** The biological synapse changes its conductance by activating/deactivating ion channels between the membrane and the synaptic junction when the action potential arrives from pre-synaptic and post-synaptic neurons coherently, while the oxide synaptic device changes its resistance by generation and migration of the oxygen vacancies when the programming voltage pulse that is larger than the threshold is applied. The neural network is emulated by the cross-point oxide synaptic device array.

The mechanism of resistive switching phenomenon in oxides has been widely attributed to the formation/rupture of the nanoscale conductive filaments which may consist of oxygen vacancies (Kown et al., 2010; Yang et al., 2012b). Figure 1 shows an analogy between the biological synapse and the artificial oxide synaptic device: the biological synapse changes its conductance by activating/deactivating ion channels between the membrane and the synaptic junction when the action potential arrives from pre-synaptic and post-synaptic neurons coherently, while the oxide synaptic device changes its resistance by generation and migration of the oxygen vacancies when the programming voltage pulse that is larger than the threshold is applied. The transition from off-state to on-state is called SET, while the transition from on-state to off-state is called RESET. During the SET, a conductive filament is formed connecting both electrodes. During the RESET, a conductive filament is ruptured and a tunneling gap is formed between one electrode and the residual filament. The variation in the tunneling gap distance results in the multilevel resistance states. The SET transition is typically abrupt due to the positive feedback between the speed of filament growth and the increase of temperature caused by the current rise (more Joule-heating) (Yu et al., 2011b). On the other hand, the RESET transition is typically gradual due to the negative feedback between the speed of filament dissolution and the decrease of temperature caused by the current drop (less Joule heating) (Yu et al., 2011b). For the oxide synaptic device, the SET transition emulates the biological potentiation process and the RESET transition emulates the biological depression process. Since the gradual RESET transition can provide multiple intermediate states, we define the learning with RESET-only as depression-only rule. In the previous work (Yu et al., 2012), we reported an analog synapse utilizing the depression-only learning rule for competitive learning. The reason why we only utilized the depression is that the RESET transition offers hundreds of states while the SET transition only offers binary states. It is believed that the analog synapse generally outperforms the binary synapse for neuromorphic computing because a limited number of synaptic states dramatically reduce the storage capacity of an artificial neural network (Senn and Fusi, 2005). If the synaptic strength cannot be changed by an arbitrarily small amount as in the case of the binary synapse, the newly learned patterns quickly overwrite the previously learned ones, thus the storage capacity is limited. This problem can be overcome by a stochastic learning rule that changes only a small fraction of synapses randomly chosen at each training cycle (Senn and Fusi, 2005). How can this random choice be realized in an oxide binary synaptic device without increasing the complexity of the CMOS neuron circuit design? Recently, Suri et al. (2012b) demonstrated a probabilistic switching in conductive bridge random access memory, which inspired implementing a stochastic learning rule for neuromorphic applications. In this work, we demonstrate that the SET transition of the oxide synaptic device becomes probabilistic under a weak programming condition (applying a smaller voltage than the nominal switching voltage), thus we propose utilizing such switching variability to realize the stochastic learning rule in the binary synapse. The stochastic SET transition was statistically measured and modeled for the oxide synaptic device. Then the system performance metrics on orientation classification function were compared between the analog synapse utilizing the depression-only learning and the binary synapse utilizing the stochastic learning. The comparison shows that with the same network storage capacity, the orientation selectivity of the system with the binary synapse is a bit higher than that of the system with the analog synapse, although the total energy consumption of the system with the binary synapse is larger than that of the system with the analog synapse. This result suggests the feasibility of using the binary synapse for neurormorphic computing. The use of binary synapse opens up new opportunities because it relaxes the constraints to engineer continuous multilevel intermediate states and widens the material choice for the synaptic device design.

## Electrical characterization of oxide synaptic device

**Figure 2:**
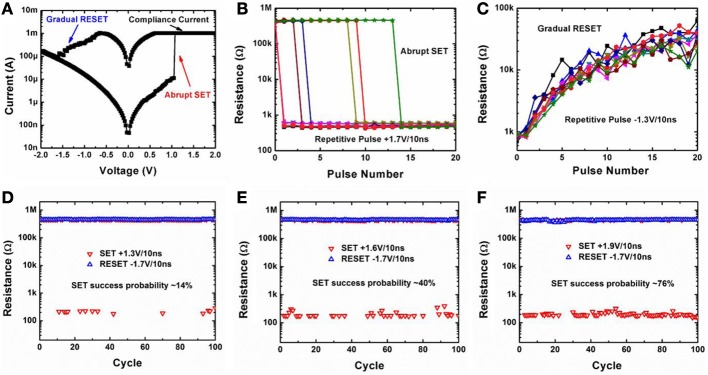
**(A)** Measured DC I-V switching characteristics of the oxide synaptic device. Abrupt SET transition and gradual RESET transition is observed. **(B)** Measured abrupt SET transition starting from the off-state (~500 kΩ) by repetitive SET pulses (+1.7 V/10 ns), in which case the device functions as a binary synapse. **(C)** Measured gradual RESET transition starting from the on-state (~500 Ω) by repetitive RESET pulses (−1.3 V/10 ns), in which case the device functions as an analog synapse. Results from 10 independent testing runs are shown. **(D–F)** Measured SET/RESET continuous cycling with different SET pulse amplitudes +1.3 V/10 ns, +1.6 V/10 ns, +1.9 V/ 10 ns, respectively. With the increase of SET pulse amplitude, the SET success probability increases as well. The SET transition becomes stochastic under weak programming condition, thus a stochastic learning rule can be utilized in such binary synapse. All the data in this figure were obtained from a single device that is representative of the devices measured.

Oxide synaptic device based on HfO_*x*_/TiO_*x*_/HfO_*x*_/TiO_*x*_ stack (from bottom to top) were fabricated (Fang et al., 2011). First, we characterized the switching characteristics of the oxide synaptic device in both DC and pulse programming mode. Figure 2A shows the measured DC I-V switching curve of the fabricated device. The device is forming-free (Fang et al., 2011) which means that no large voltage is required to trigger the subsequent switching behaviors and the as-fabricated device resistance is around 500 kΩ off-state. The SET transition occurs around +1 V with an abrupt jump of current to the compliance current level (1 mA). The RESET transition starts from −0.7 V to −1.6V with a gradual decrease of current. The abrupt SET transition and gradual RESET transition is also observable in the pulse switching mode, as shown in Figures 2B,C. When the repetitive SET pulse (+1.7 V/10 ns) was applied to the device in the off-state, the potentiation process is abrupt and only two states can be obtained (~500 kΩ and ~500 Ω). In contrast, when repetitive RESET pulse (−1.3 V/10 ns) was applied to the device in the on-state, the depression process is gradual and multilevel intermediate states can be obtained. Thus, the device can serve as an analog synapse with the depression process. We optimized the RESET condition (e.g., −1.1 V/10 ns) as the analog synapse for the depression-only learning rule (Yu et al., 2012) (see the Appendix). Interestingly, we found that although the SET transition is abrupt, it becomes probabilistic under a weak programming condition. Figures 2D–F shows the measured SET/RESET continuous cycling with different SET pulse amplitudes (+1.3 V/10 ns, +1.6 V/10 ns, +1.9 V/10 ns, respectively).

It is seen that with decrease of the SET pulse amplitude, the SET success probability decreases as well. The resistive switching is inherently stochastic due to the randomness of the oxygen vacancy's generation and migration as suggested by the Kinetic Monte Carlo simulation in (Yu et al., 2011b). The remarkable switching parameter variability is a well-known technical challenge for the oxide based resistive switching memory array design and substantial research efforts were spent to reduce the variability (Yu et al., 2013). Here we make use of the nominal disadvantage (from a digital memory perspective) to realize the stochastic learning rule for the binary synapse.

**Figure 3:**
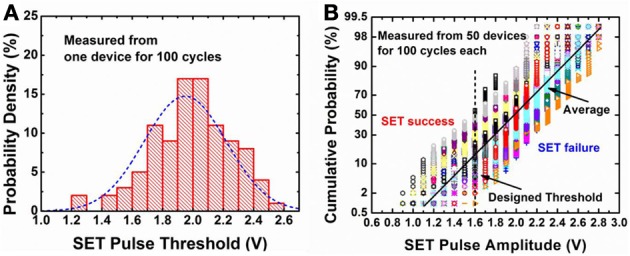
**Measured statistical distribution of pulse amplitude required for triggering the SET switching from the off-state.** In **(A)** the probability of SET switching is measured for one representative device for 100 cycles. In each cycle, a strong RESET pulse (−1.9 V/10 ns) to was applied to achieve a complete off-state (~500 kΩ), then a weak SET pulse with amplitudes from +0.6 V to +3 V (with linearly spaced steps with increasing amplitude) with a 10 ns width was applied to determine the switching probability. Such cycle was repeated for 100 cycles for each device. In **(B)**, 50 different devices on the wafer were measured in the way as described for **(A)**. The data for these 50 devices are presented in **(B)** with one type of symbol in the figure representing the data from one device. The y-axis in **(B)** is scaled to be Gaussian, thus a straight line in this plot indicates a Gaussian distribution. If the applied pulse amplitude is 1.6 V, then on average, around 12% of the SET trials will be successful.

To obtain the statistics for both cycle-to-cycle variation and device-to-device variation, we measured the pulse amplitudes required for triggering the SET transition (with fixed 10 ns pulse width) during 100 cycles in one device and repeated such testing for 50 different devices. Figure 3 shows the measured statistical distribution: **(A)** for a particular device, the pulse amplitude for a successful SET operation roughly follows a Gaussian distribution with a standard deviation about 0.3 V; **(B)** across various devices, the medium pulse amplitude for a successful SET operation is centered around 1.95 V with a standard deviation about 0.15 V. If we design the pulse amplitude applied to the device to be 1.6 V, then on average, around 12% SET trials will be successful. Certainly, due to device-to-device variation, some device may have success probability higher than 12%, while others may have success probability lower than 12%. Nevertheless, the SET transition becomes probabilistic under this weak programming condition. The origin of the stochastic SET switching is worth discussion. We suggests there is some sort of SET threshold (but not well-defined) associated with the internal state of the device (e.g., the tunneling gap distance or oxygen vacancy distribution). After each RESET pulse applied on the device, the internal state is disturbed somehow. Even if the device is in the same resistance states in the off-state, internally the oxygen vacancy distribution may be different. Therefore, there may be another new SET threshold (but not well-defined) in the next SET cycle if the device is under disturbance pulse at the current cycle. The indeterministic SET threshold is resulted from the variation of the internal state of the device. The purpose of the testing in Figure 3 is to measure such SET threshold distribution after the disturbance by a RESET pulse. Further detailed study on the physical origin of the stochastic SET switching is needed, and the Kinetic Monte Carlo simulation in (Yu et al., 2011b) may offer deeper insights on this issue.

In this work, we use a weak SET condition (e.g., +1.6 V/10 ns) with a strong RESET condition (e.g., −1.9 V/10 ns) for a stochastic learning rule. A RESET pulse larger than −1.6 V/10 ns can be considered as strong programming condition for achieving the complete off-state with a single pulse (Yu et al., 2012). Here a strong RESET is needed to switch the device to a complete off-state to avoid any unintentional switching under a weak SET programming condition in the next cycle. Due to the non-volatility of the resistive switching in the oxide synaptic device (which means the resistance states should be stable without applying voltage stress), we would expect that the probability of SET would not depend on the neuron firing rate, and the stability of the intermediate states is not a big concern. However, these issues are worth further study.

## Simulation of winner-take-all network

**Figure 4:**
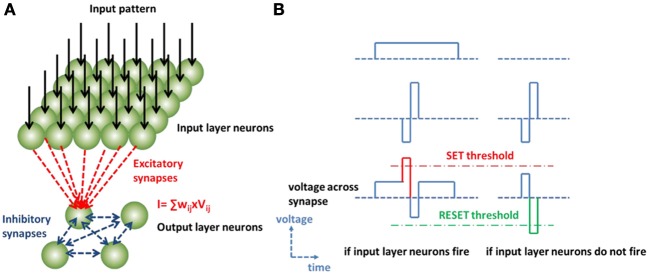
**(A)** Neuromorphic system based on winner-take-all neural network. In the system-level simulation, 32 × 32 neurons in the input layer are connected with 2 × 2 neurons in the output layer through 4096 oxide based excitatory synaptic devices. Every neuron in the output layer also connects to one another through inhibitory synapses based on fixed resistors. **(B)** The spiking scheme for binary synapse with stochastic learning: the pre-synaptic forward spike from the input layer neuron is designed to be a long but small positive pulse (e.g., +0.8 V/500 ns), the post-synaptic backward spike is designed to be a short two-phase pulse with a small negative pulse (e.g., −0.8 V/10 ns) followed by a large positive pulse (e.g., +1.9 V/10 ns). If both the input layer neuron and the output layer neuron fire, the synapse faces an actual SET programming pulse (e.g., +1.6 V/10 ns); if only the output layer neuron fires, the synapse faces an actual RESET programming pulse (e.g., −1.9 V/10 ns). Thus, the synapse conductance map between the input layer and the output layer tends to mimic the input pattern light intensity.

To validate the stochastic learning rule with oxide binary synaptic device, we perform a simulation of a two-layer winner-take-all neural network as a toy model. Figure 4A shows the network architecture implemented by integrate-and-fire neurons and oxide synaptic devices: every neuron in the output layer connects with all the neurons in the input layer through excitatory synapses based on the oxide synaptic devices. Every neuron in the output layer also connects to one another through inhibitory synapses based on fixed resistors. The unsupervised competitive learning algorithm allows such two-layer network to perform the orientation classification function (Zamarreño-Ramos et al., 2011). A spiking scheme for implementing the unsupervised competitive learning algorithm in the binary synapse can be designed in Figure 4B: the input layer neurons fire according to the light intensity of the input pattern; if the light intensity exceeds the neuron firing threshold, the neurons send a relatively long but small positive pulse to all the output layer neurons through the excitatory synapses. The output layer neurons sum and integrate the input currents on the membrane capacitor independently, and the one with the largest input current fires first (becomes the “winner”), then it discharges the membrane capacitor of all the other output layer neurons and prevent them from firing (“takes all”) through the inhibitory synapses. Meanwhile this winner neuron sends a short two-phase pulse with a small negative pulse followed by a large positive pulse back to all the input layer neurons. Thus, the excitatory synapse strength gets modified according to the input pattern: if both the input layer neuron and the output layer neuron fire, the synapse may face an actual SET programming pulse larger than the threshold; if only the output layer neuron fires, the synapse may face an actual RESET programming pulse larger than the threshold. Thus, the synapse conductance map between the input layer and the output layer tends to mimic the input pattern light intensity. Since the SET transition is probabilistic under a weak programming condition, the update of the synapse conductance map is an incremental process. After a certain number of training images, a self-organized conductance map emerges. In the following simulation, 32 × 32 neurons in the input layer are used and 2 × 2 neurons in the output layer are used. Thus, there are 4096 oxide synaptic devices between the two layers. During the training, 200 gray-scale images of a 2D Gaussian bar with random orientation were presented to the input layer neurons. These orientations have a non-uniform distribution (centered at 0, 45, 90, and 135° with a standard deviation of 7.5°). The target of the network is to converge at these 4 dominate orientations.

**Figure 5:**
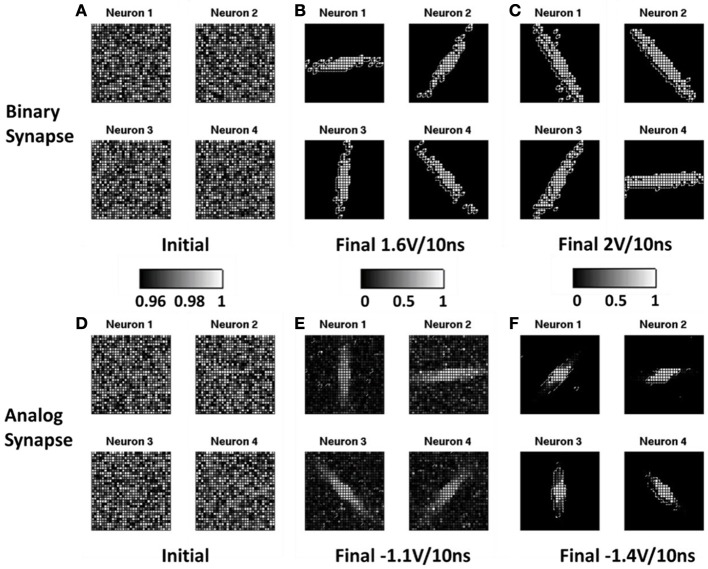
**Simulated normalized conductance map between the input layer neurons and the output layer neurons utilizing binary synapse with stochastic learning (A–C) and analog synapse with depression-only learning (D–F).** The normalization is done with respect to a reference that is the highest conductance in the synapse array before the training. Initially, the resistances of all the oxide synaptic devices were randomized with a distribution centered at on-state for binary synapse **(A)** and for analog synapse **(D)**. After the training, the resistances diverge. With appropriate programming condition, the 4 distinct orientations emerge, e.g., for binary synapse using +1.6 V/10 ns SET pulse **(B)** and for analog synapse using −1.1 V/10 ns RESET pulse **(E)**. If the programming condition not optimized, only 3 distinct orientations emerge, e.g., for binary synapse using +2 V/10 ns SET pulse **(C)** and for analog synapse using −1.4 V/10 ns RESET pulse **(F)**.

**Figure 6:**
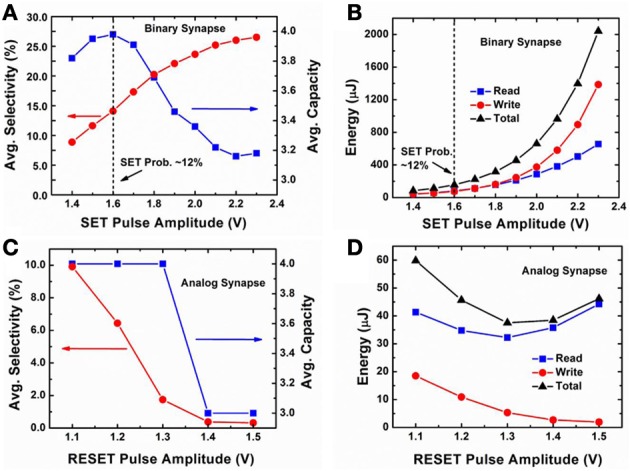
**Simulated system performance metrics as a function of programming conditions.** Network orientation selectivity and orientation storage capacity for binary synapse in **(A)** and for analog synapse in **(C)**; Energy consumption of the synaptic devices during the whole training (200 training images) for binary synapse in **(B)** and for analog synapse in **(D)**. The average values through 100 independent simulation runs are shown. +1.6 V/10 ns is chosen as the optimized programming condition for binary synapse, which corresponds to a SET success probability ~12% on average. And −1.1 V/10 ns is chosen as the optimized programming condition for analog synapse.

Figure 5 shows the evolution of the normalized conductance map between the input layer neurons and the output layer neurons for the binary synapse with stochastic learning **(A–C)** and the analog synapse with depression-only learning **(D–F)**. Initially, the resistances of all the oxide synaptic devices were randomized with a distribution centered at an on-state (~500 Ω), as shown in Figure 5A for the binary synapse and in Figure 5D for the analog synapse. After the training, the resistances split into groups of the on-state and the off-state. With appropriate programming condition, 4 distinct orientations emerge, as shown in Figure 5B for the binary synapse using +1.6 V/10 ns SET pulse and in Figure 5E for the analog synapse using −1.1 V/10 ns RESET pulse. It is noted that for the analog synapse, there are many noisy pixels caused by the intermediate states. If the programming condition not optimized, only 3 distinct orientations emerge, as shown in Figure 5C for the binary synapse using +2 V/10 ns SET pulse and in Figure 5F for the analog synapse using −1.4 V/10 ns RESET pulse. To compare the system performance between the binary synapse and the analog synapse, three metrics are used: (1) the orientation selectivity defined as the contrast of the output layer neuron's response intensity to the 1st preferred orientation over the 2nd preferred orientation; (2) the orientation storage capacity defined as the number of distinct orientations stored in the output layer (ideally, 4 distinct orientations will be detected); (3) the energy consumed on the synaptic devices during the whole training, including the read energy for summing the current through the synapses and the write energy for programming the synapses. Figure 6 shows the average values of these metrics as a function of programming conditions for the system with the binary synapse **(A–B)** and the system with the analog synapse **(C–D)** through 100 independent simulation runs (with the same training data sets). The effect of using random training data sets remains for further study. The trends in Figure 6 can be explained as follows: for the binary synapse, increasing the SET pulse amplitude means increasing the SET success probability. As a result, the selectivity increases because more pixels are switched to “white” and the contrast is improved. The orientation storage capacity can achieve the maximum value 4 at 1.6 V, thus +1.6 V/10 ns is chosen as the optimized programming condition for the binary synapse, which corresponds to a SET success probability ~12% on average. The loss of the orientation storage capacity below 1.6 V SET pulse amplitude is due to insufficient SET success probability, which limits the ability of the network to learn sufficient patterns for a fixed (limited) set of training images (200 images in this case). On the other hand, the rapid drop of the orientation storage capacity beyond 1.6 V SET pulse amplitude is due to excessive SET success probability, which hastens the network's forgetting process (overwriting the learned patterns too frequently), thus only the final patterns are remembered (see Figure 5C as an example). The total energy consumption (including the read and write energy) increases with the increase of the SET pulse amplitude. The energy consumption roughly follows the relationship ~ *E* = (*V*^2^/*R*) × *t*. For the analog synapse, increasing the RESET amplitude means that the RESET transition becomes less gradual and fewer intermediate states are available (Yu et al., 2012). As a result, both the selectivity and the orientation storage capacity decreases with increasing RESET pulse amplitude (see Figure 5F as an example). Therefore, in general, the lower the amplitude, the better. Here −1.1 V/10 ns is chosen as the optimized programming condition for the analog synapse because the pulses smaller than −1.1 V almost could not affect the resistance (Yu et al., 2012). Under depression-only mode, the learning becomes saturated as the devices quickly RESET to the completely off-state if the number of possible intermediate states are insufficient. The write energy consumption decreases with the increase of RESET pulse amplitude since the learning saturates faster. The read energy has a turning point due to the competing trends of increasing voltage and increasing resistance in the relationship ~ *E* = *V*^2^/*R* × *t*. At the optimized programming condition for the binary synapse and the analog synapse, respectively, the same full network storage capacity of 100% is achievable, the selectivity of the binary synapse is 14.1% and that of the analog synapse is 9.9%, and the total energy consumption of the binary synapse is 156 μJ and the that of the analog synapse is 60 μJ. The feasibility of the stochastic learning with the binary synapse is demonstrated through this system-level simulation.

## Conclusion

In summary, we demonstrate that the SET transition of oxide synaptic device becomes probabilistic under a weak programming condition. The switching variability can be utilized to implement a stochastic learning rule. A simulation of winner-take-all network was performed for orientation classification function, showing comparable system performance between the analog synapse utilizing the depression-only learning and the binary synapse utilizing the stochastic learning. The significance of this demonstration is that it opens up new opportunities for a variety of material and device choices for implementing neuromorphic computing in the hardware. Further studies on the physical origin of such stochastic SET process is helpful, and the simulation beyond this winner-take-all toy-model is necessary to evaluate the effectiveness of such stochastic learning algorithms on real-world problems.

## Methods

### Device fabrication

Fifty nanometer Pt bottom electrode (with 20 nm Ti adhesion layer) was first deposited on 8-inch Si substrate by electron beam evaporation. Four nanometer HfO_*x*_ was deposited by reactive sputtering in Ar and O_2_ ambient, and then 2 nm TiO_*x*_ was prepared by oxidation of Ti thin film. These two processes were repeated to form the 12 nm four-layer oxide stack of HfO_*x*_/TiO_*x*_/HfO_*x*_/TiO_*x*_ (from bottom to top). Then a 50 nm TiN top electrode was deposited by reactive sputtering and patterned by photo-lithography with the 5 μm by 5 μm pad size. Finally, dry etch was done to isolate the cells on the wafer. Materials characterization techniques such as cross-sectional transmission electron microscopy (TEM) and energy-dispersive X-ray (EDX) spectroscopy were employed to study the cross-sectional morphology and elemental spatial profile, which were reported in (Fang et al., 2011).

### Device electrical measurement

Keithley 4200 semiconductor parameter analyzer and Agilent 81150A pulse generator were used for DC and pulse electrical measurements, respectively. In all the measurements, the voltage was applied to the top electrode (TiN) while the bottom electrode (Pt) was grounded. The resistance state were all measured with a read voltage of 0.1 V.

The experimental protocol for the measurement in Figure 2 is as follows: for a particular device, **(A)** DC I-V sweep was performed from 0 V → + 2 V → 0 V →− 2 V → 0 V. During SET, a 1 mA compliance current was enforced for the prevention of permanent breakdown of the device. **(B)** SET transition starting from the off-state (~500 kΩ) by repetitive SET pulses (+1.7 V/10 ns). Ten independent testing runs were performed. **(C)** RESET transition starting from the on-state (~500 Ω) by repetitive RESET pulses (−1.3 V/10 ns). Ten independent testing runs were performed. **(D–E)** SET/RESET 100 times continuous cycling with a fixed RESET pulse (−1.7 V/10 ns) but different SET pulse amplitudes (+1.3 V/10 ns, +1.6 V/10 ns, +1.9 V/10 ns), respectively.

The experimental protocol for the measurement in Figure 3 is as follows: 50 different devices on the wafer were measured, and in each device 100 cycles were measured. In each cycle, a strong RESET pulse (−1.9 V/10 ns) to was applied to achieve a complete off-state, then a weak SET pulse linearly ramping from +0.6 V to +3 V with a 10 ns width was applied to determine the switching threshold voltage; and such cycle were repeated for 100 cycles for each device. The purpose of the testing in Figure 3 is to measure such SET threshold distribution after the disturbance by a RESET pulse. Therefore, no intermediate RESET pulse in between were applied when linearly ramping the SET voltage amplitudes. However, this measurement protocol may introduce some systematic bias, e.g., some shift of the SET threshold distribution toward lower amplitudes due to the accumulation effect of the pulses. But such shift is expected to be insignificant considering the exponential dependence of the oxygen vacancy generation rate on the applied voltage (Yu et al., 2011b).

### Winner-take-all network simulation

According to the measurement results in Figure 3, the stochastic switching behavior is modeled as follows: the cycle-to-cycle variation of the binary synapse is modeled as a Gaussian distribution of the threshold SET pulse amplitude (with a standard deviation 0.3 V); and the median value of the Gaussian distribution shifts from device to device, reflecting the device-to-device variation, which is modeled as a Gaussian distribution centered around 1.95 V with a standard deviation 0.15 V. Then this model was implemented in the following simulation in such a way: each synapse in the network is randomly assigned with a SET threshold with different thresholds following the Gaussian distribution centered around 1.95 V with a standard deviation 0.15 V, and different synapses have different SET threshold voltages reflecting the device-to-device variation. Then during the simulation, if a synapse is disturbed by an intermediate RESET pulse, the new SET threshold is assigned to that synapse following the Gaussian distribution with a standard deviation 0.3 V, reflecting the cycle-to-cycle variation caused by the RESET pulse disturbance on the internal state of the device. At each cycle, the actual voltage dropped on the synapse, which is designed by the spiking schemes described in Figure 4B, is compared with the SET threshold of each synapse in the network: if the actual voltage is larger than the SET threshold at that particular cycle, the synapse is switched from off-state to on-state, otherwise, it remains off-state. If the synapse sees a RESET pulse, it is unconditionally switched from on-state to off-state since the RESET pulse is designed to be a strong RESET pulse (See Figure 4B).

The two-layer winner-take-all neural network is simulated in MATLAB with the above stochastic binary synapse model and a typical integrate-and-fire neuron model.

$$C_{m}\frac{dV}{dt}=I-\frac{V}{R_{L}}$$

The neuron firing threshold is set to be 1 V. The leaky resistor is set to be 1 MΩ, and the membrane capacitor is 1 pF, thus the decay time constant of the membrane voltage is set to be 1 μs. The pre-synaptic forward spike from the input layer neuron is designed to be a positive pulse (e.g., +0.8 V/500 ns) that is half the amplitude of the actual SET programming pulse, the post-synaptic backward spike from the output layer neuron is designed to be a negative pulse (e.g., −0.8 V/10 ns) that is half the amplitude of the actual SET programming pulse followed by a positive constant pulse (e.g., +1.9 V/10 ns), see Figure 4B for an illustration. Changing the pulse amplitude also affects the synaptic transmission since the input current of the output layer neurons is proportional to the spike pulse amplitude of the input layer neurons.

Initially, the resistances of all the oxide synaptic devices were randomized with a distribution centered at the on-state (~500 Ω). During the training, 200 gray-scale testing images with 32 × 32 pixels were presented into the input layer neurons. The input patterns have the shape of a 2D Gaussian bar with random orientation. The decay length of the 2D Gaussian bar in longitude direction is 16 pixels and the decay length in latitude direction is 4 pixels. The input stimuli are synchronized. The input layer neuron fires if the relative intensity is larger than 0.5. These 200 test images have a non-uniform distribution in 4 orientations (centered at 0, 45, 90, and 135° with a standard deviation of 7.5°). The energy consumed on all the synapses in the network was calculated during the training.

When the training was completed after 200 training images by definition, standard images of 2D Gaussian bar in 24 different orientations (0° to 180 with a step of 7.5°) were used for testing the orientation selectivity of the network. The orientation selectivity was calculated as the contrast of the output layer neuron's response intensity to the 1st preferred orientation over the response intensity to the 2nd preferred orientations. The orientation storage capacity was defined as the number of distinct orientations in the output layer.

### Conflict of interest statement

The authors declare that the research was conducted in the absence of any commercial or financial relationships that could be construed as a potential conflict of interest.

## Figures and Tables

**Figure A1:**
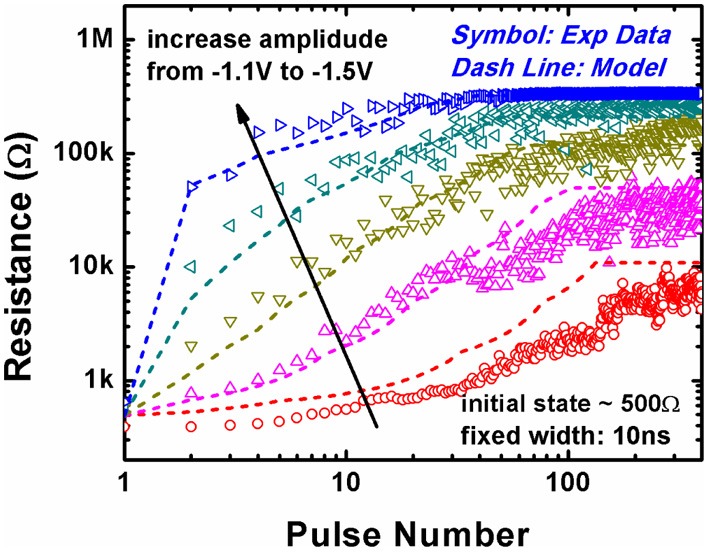
**Measured gradual RESET transition starting from ~500 Ω by hundreds of RESET pulses.** The pulse amplitude was varied, and the parameters of the compact model were fitted.

**Figure A2:**
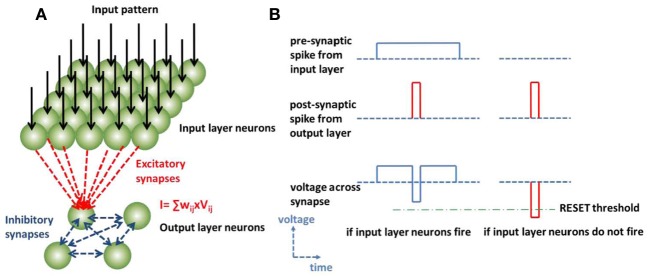
**The spiking scheme for the analog synapse utilizing the depression-only learning**.

## Appendix

### The gradual reset transition as an analog synapse

The oxide synaptic device has a gradual RESET transition. As reported in Yu et al. (2012) of the main text, multilevel intermediate states can be obtained by hundreds of RESET pulses. Figure A1 shows the RESET transition starting from on-state (~500 Ω) with different RESET pulse amplitudes: a lower amplitude leads to a more gradual RESET transition than a higher amplitude does. Therefore, −1.1 V/10 ns is chosen as a preferred programming condition for analog synapse. For system-level simulation, a compact model of filament dissolution was developed to capture this gradual RESET transition. The model was fitted with the experimental data, and the details of the model can be found in Yu et al. (2012).

### The spiking scheme for analog synapse with depression-only learning

Since the analog synaptic behavior can only be obtained in the gradual RESET transition (not in the abrupt SET transition), a spiking scheme for depression-only learning is designed as following in Figure A2. It is similar as the scheme for binary synapse showed in Figure 5B of the main text: the pre-synaptic forward spike from the input layer neuron is designed to be positive pulse with half amplitude of the actual RESET programming pulse (e.g., +0.55 V/500 ns), the post-synaptic backward spike is designed to be a positive pulse with amplitude of the actual RESET programming pulse (e.g., +1.1 V/10 ns). Therefore, if both the input layer neuron and the output layer neuron fire, the synapse does not face any programming pulse due to the cancelling effect of the forward spike and backward spike; if only the output layer neuron fires, the synapse faces an actual RESET programming pulse (e.g., −1.1 V/10 ns). In this way, the depression-only learning for analog synapse is realized.
